# Alienation Appraisals Mediate the Relationships between Childhood Trauma and Multiple Markers of Posttraumatic Stress

**DOI:** 10.1007/s40653-018-0220-1

**Published:** 2018-06-18

**Authors:** Ryan Mitchell, Donncha Hanna, Kate Brennan, David Curran, Brian McDermott, Margaret Ryan, Kelly Craig, Emma McCullough, Paulette Wallace, Kevin F. W. Dyer

**Affiliations:** 1 School of Psychology, Queen’s University Belfast, Belfast, BT9 5BN Northern Ireland; 2 Trauma Resource Centre, Belfast HSC Trust, Belfast, BT14 7GB Northern Ireland; 3 Psychological Therapies Service, Holywell Hospital, Northern HSC Trust, Antrim, BT41 2RJ Northern Ireland

**Keywords:** Trauma appraisals, Posttraumatic stress, Depression, Childhood trauma, Cumulative trauma, Mediation, Alienation

## Abstract

Rates of posttraumatic stress are elevated in individuals who have experienced childhood and/or cumulative trauma, and trauma appraisals have been suggested as a possible mediator of this effect. This study tested the proposed mediating role of trauma appraisals between both childhood and cumulative trauma, and two markers of trauma-related distress; posttraumatic stress and depression. Mediation models were developed and tested with data collected from a sample of trauma-exposed, treatment receiving adults (*N* = 106). Trauma appraisals fully mediated relationships between childhood trauma and PTSD/depression. Appraisals also mediated the relationships between cumulative trauma and depression. When appraisal subscales were simultaneously entered, alienation appraisals were the only significant mediator of these relationships. The study found support for the proposed mediating role of trauma appraisals between different forms of trauma and trauma related distress. Alienation appraisals were particularly emphasised.

Psychopathological sequalae of traumatic experiences have most commonly been understood in line with the Diagnostic and Statistical Manual (DSM) diagnosis of Posttraumatic Stress Disorder (PTSD). PTSD has a lifetime prevalence of 8.3% (Kilpatrick et al. 2013), and has a large negative effect on quality of life (Giacco et al. 2013). Psychological comorbidity in PTSD is the “rule rather than the exception” (Brady et al. 2000, p.22), with a large majority of individuals presenting with one or more concurrently diagnosable conditions, and depression presenting as the most common comorbid difficulty (Brady et al. 2000).

A review by Rosen and Lilienfeld (2008) identified that whilst there is some supportive evidence for a ‘dose-response’ relationship between frequency/severity of trauma and PTSD (Brewin et al. 2000; Galea et al. 2002), some studies have not supported this relationship (Bowman 1999). In addition, Rosen and Lilienfeld (2008) noted that a majority of people do not develop PTSD after experiencing DSM-IV criterion A traumatic events, and that the character of the event itself explains less variance in clinical outcomes than other psychological and social variables. Given the variability in results regarding ‘dose-response’ effects, it has been suggested that the range of experienced trauma types, rather than frequency of experienced traumas, is more relevant in the development of symptoms (Gamache-Martin et al. 2013). Evidence suggests that experiencing cumulative trauma, defined in Gamache-Martin et al. (2013) as exposure to a range of different *types* of potentially traumatic events, is associated with more severe and enduring mental and physical health problems including obesity, chronic pain, depression, dissociation, PTSD symptoms and substance abuse (Gamache-Martin et al. 2013; Anda et al. 2006; Goldberg et al. 1999). Moreover, Wilker et al. (2015) found that cumulative trauma defined in this way was a stronger predictor of lifetime PTSD than frequency of experienced trauma.

Childhood abuse and other forms of childhood adversity have also been posited as risk factors for PTSD in adulthood (Clark and Beck 2011). Childhood maltreatment asserts a moderate to large negative impact on psychiatric symptoms in general (Pietrek et al. 2013), and effect sizes are very large for exposure to multiple forms of abuse (Teicher et al. 2006). In a large-scale review, Brewin et al. (2000) found that childhood adversity was a consistent predictor of later PTSD, although the magnitude of this effect was heterogeneous across studies. Further, they found that childhood abuse was a more uniform predictor of PTSD severity across studies than childhood adversity without abuse, suggesting that abuse and adversity in childhood both increase the risk of PTSD in adulthood, with abuse being particularly toxic.

In Ehlers and Clark’s (2000) model of PTSD, they hypothesise that *prior experiences* of childhood abuse or trauma may heighten negative appraisals of trauma in adulthood via a reactivation of memories of early trauma, leading to negative interpretations in line with these distressing memories. Consistent with this, Babcock and DePrince (2012) demonstrated that childhood betrayal trauma, i.e. trauma perpetrated by a trusted other, predicted self-blame for intimate partner abuse in adulthood. In order to better understand the mechanism behind the influence of cumulative trauma and childhood trauma on trauma related distress, further evaluation of appraisals as potential cognitive mediating factors may prove useful.

Cognitive theories of posttraumatic stress disorder suggest that maladaptive appraisals of the traumatic event and its sequalae mediate the relationship between the event itself and trauma-related distress (Ehlers and Clark 2000; Foa and Rothbaum 1998). DePrince et al. (2010) define appraisals as “*people’s assessments of their thoughts, feelings and behaviours” (p.276)*, subsequent to a traumatic event. Ehlers and Clark (2000) suggest that characteristics of the traumatic event(s) and prior experiences influence the manner in which the trauma and its sequalae are appraised, which in turn influence the sense of current threat, current emotions, and strategies intended to control these. Maladaptive appraisals have previously been measured using the posttraumatic cognitions inventory (PTCI; Foa et al. 1999), which comprises three subscales including negative cognitions about the self, negative cognitions about the world, and self-blame. Using the PTCI, maladaptive appraisals have been shown to correlate with PTSD symptoms (Laposa and Alden 2003), and predict the development of symptoms over time (Dunmore et al. 2001; Ehring et al. 2008). However, the three subscales of the PTCI are broad in scope, and it is possible that additional specific appraisals may be relevant. To this end, the recent development of the trauma appraisal questionnaire (TAQ), based on item generation via 72 interviews with a diverse community sample of trauma exposed individuals has implicated a range of further specific appraisal types in posttraumatic stress (DePrince et al. 2010). DePrince et al. (2010) developed the TAQ as a comprehensive measure of trauma appraisals, which evidenced subscales of betrayal, self-blame, fear, alienation, anger and shame. They found strong support for the roles of the novel appraisals of alienation and betrayal in causing trauma-related distress in the form of PTSD symptoms, dissociation and depression (DePrince et al. 2011).

Appraisals have been shown to mediate the relationship between posttraumatic symptoms immediately following a motor vehicle accident, and at follow up (Meiser-Stedman et al. 2009). Moreover, Barlow et al. (2017) recently found that trauma appraisals fully mediated the relationship between childhood abuse and adult symptoms of PTSD in a student sample. The current study extended this work by testing trauma appraisals as mediators of the relationships between childhood trauma/cumulative trauma, and two measures of trauma-related distress, in a sample of treatment receiving individuals.

The study tested the proposed mediating role of trauma appraisals in predicting two different forms of trauma related distress/functional impairment, i.e. PTSD and depression. It is a core feature of cognitive models of PTSD that appraisals are crucial in the development of adverse reactions to trauma, and the present research provides a direct test of these highly influential models. The study also tested two predictions put forward in the seminal Ehlers and Clark (2000) paper, by selecting theoretically and empirically supported predictor variables of *prior experiences* (conceptualised as childhood trauma), and *characteristics of the trauma* (conceptualised as cumulative trauma). In addition, identification of the *specific* appraisals that mediate the relationship between traumatic experiences and traumatic stress may lead to more focused intervention protocols.

The aim of the current study was therefore to test four mediation models. Model 1 suggested the relationship between cumulative trauma and posttraumatic stress symptoms would be mediated by appraisals of trauma. Model 2 suggested the relationship between cumulative trauma and depression would be mediated by appraisals of trauma. Model 3 suggested the relationship between childhood trauma and posttraumatic stress symptoms would be mediated by appraisals of trauma, and model 4 suggested the relationship between childhood trauma and depression would be mediated by appraisals of trauma.

## Method

### Participants

Participants (*N* = 106) were recruited from four sites across two health trusts in Northern Ireland. Sites included a psychological therapies service (*N* = 26), a community addictions service (*N* = 13), an inpatient addictions ward (*N* = 14), and a specialist community trauma service (*N* = 53). All three services provide psychological interventions for trauma. Participants were included if they were over 18 years old, and had experienced one or more DSM-5 criterion A traumatic events. Participants were excluded if they had a degenerative neurological condition. Therapists at each site identified suitable participants who were subsequently invited to participate by the authors, if they consented to be contacted regarding the study. Ethical approval was granted by ORECNI.

### Measures

#### Posttraumatic Stress Diagnostic Scale (PDS; Foa et al. 1997)

This is a four-part measure of trauma and posttraumatic stress. Part 1 is a checklist of traumatic experiences corresponding to DSM criterion A stressors. Part 1 formed the cumulative trauma variable in this study, by summing the total number of different trauma types experienced across the lifespan. Potential trauma types included; accident, disaster, non-sexual assault/someone you know, non-sexual assault/stranger, sexual assault/someone you know, sexual assault/stranger, combat, sexual contact under 18 with someone 5 or more years older, imprisonment, torture, life-threatening illness, and ‘other’. Part 2 requires participants to indicate the worst event they have experienced in part 1. Part 3 assesses severity of posttraumatic stress symptoms, constituting the continuous variable of posttraumatic stress symptoms in this study. Part 4 assesses functional impairment.

Internal consistency across subscales has been reported as 0.94–.97 (Coffey et al. 1998), test-retest reliability for the full scale was .74 (Foa et al. 1993), and the measure demonstrates a range of good validities (Foa et al. 1997). Internal consistency in the current study was .92.

#### Childhood Trauma Questionnaire (CTQ; Bernstein and Fink 1998)

The CTQ is a 28-item measure that assesses childhood trauma, focusing on abuse and neglect. The measure comprises five subscales, including emotional abuse, physical abuse, sexual abuse, emotional neglect and physical neglect. The CTQ also incorporates a minimization scale that identifies potential underreporting of abuse/neglect. Test-retest reliability is good (Bernstein and Fink 1998). The CTQ total score was the variable of interest in this study. Evidence of good reliability has been found in clinical and community samples, (Scher et al. 2001). Internal consistency for the total score in this sample was .90.

#### Trauma Appraisal Questionnaire (TAQ; DePrince et al. 2010)

This 54-item measure assesses appraisals that people make of their thoughts, feelings and behaviours subsequent to a traumatic event. It comprises 6 subscales including fear, alienation, anger, betrayal, shame, and self-blame. Items are rated on a 5 point likert scale with respect to current thoughts, feelings and experiences when participants think about their traumatic event. The items of the alienation subscale are as follows: ‘I felt lonely’, ‘There was a huge void inside me’, ‘Even though I had friends, I was still lonely’, ‘I mostly stayed to myself’, ‘I was disconnected from people’, ‘I cut myself off from other people’, ‘I couldn’t get close to people’, ‘I lost a piece of myself’, ‘My friends didn’t understand my reactions’, ‘I didn’t want to have to trust anyone’.

Across a range of samples, the subscales demonstrated excellent internal consistency (α = 0.84–.93, DePrince et al. 2010) and good test-retest reliability (*r =* .73–.88). Convergent, concurrent and discriminant validity were also excellent. In the current study internal consistency was high for the full TAQ (α = 0.96), and for the individual subscales (α = 0.84 to .90).

#### Patient Health Questionnaire (PHQ-9; Kroenke et al. 2001)

This is a brief self-report measure of depression severity. A series of 9 potential problems are presented, and participants rate the extent to which they have experienced these problems over the last 2 weeks, on a 4-point scale from ‘not at all’ to ‘nearly every day’. Construct validity is good (Kroenke et al. 2001) and the measure has demonstrated high internal consistency and test-retest reliability (Zuithoff et al. 2010). Internal consistency in this sample was high (α = .89).

### Procedure

Participants completed all measures in a single session at their usual therapy location. Measures were completed as part of a larger study, and were administered in the following order; Demographic information, Fears of Compassion Scales, PDS, TAQ, CTQ, PHQ-9, and Brief Resilience Scale. The Fears of Compassion Scales and Brief Resilience Scale were not analysed in the current study. Participants identified the worst trauma they had experienced via the PDS and were asked to complete the TAQ with respect to this event.

### Analyses

Analyses were conducted in IBM SPSS statistics version 20.0.0.1. Four one-way analyses of variance were undertaken to assess whether participants from different sites differed significantly on the predictor variables of cumulative trauma and childhood trauma, and the outcome variables of posttraumatic stress symptoms and depression. Correlations were then calculated between cumulative trauma, childhood trauma, trauma appraisal total scale and subscales, and depression. Next, a series of multiple parallel mediation models were conducted using PROCESS for SPSS (Hayes 2012). Mediation models were conducted if a significant relationship was observed between predictor and outcome variables. The initial three models had a single mediator (trauma appraisals total), with predictor variables of childhood and cumulative trauma, and outcome variables of depression and posttraumatic stress symptoms. If significant mediation was found, the models were rerun, but with all trauma appraisal subscales included as mediators to identify the most relevant appraisal types. The subsequent three models featured the same predictor and outcome variables, but had multiple mediators (all trauma appraisal subscales), permitting the calculation of the specific indirect effect of each specific appraisal type, while all others were controlled (Hayes 2013). 2000 bias corrected bootstrap samples were used to generate 95% confidence intervals and point estimates for indirect effects. Assumptions of homoscedasticity and linearity of relationships were met and there were no problems with multicollinearity.

## Results

Table 1 shows descriptive statistics for the measures used.

**Table 1:** Descriptive statistics and Cronbach’s alpha values for measures

Measures	Construct	Mean	SD	Range	Potential Range	α
TAQ	Total Appraisals	174.90	45.25	54–266	54–270	.96
	Alienation	38.24	9.94	10–50	10–50	.90
	Fear	43.46	11.83	12–60	12–60	.89
	Shame	22.99	7.78	7–35	7–35	.81
	Self-blame	25.98	10.92	10–50	10–50	.86
	Betrayal	20.97	8.80	7–35	7–35	.86
	Anger	23.67	8.70	8–40	8–40	.84
CTQ	Childhood Trauma	52.50	24.58	25–113	25–125	.90
PDS	Cumulative Trauma	4.12	2.39	1–11	0–12	N/A
	PTSD symptoms	35.30	12.00	0–51	0–51	.92
PHQ	Depression	18.46	7.91	0–30	0–36	.89

*TAQ* Trauma Appraisal Questionnaire, *CTQ* Childhood Trauma Questionnaire, *PDS* Posttraumatic Stress Diagnostic Scale, *PHQ* Patient Health Questionnaire

Table 1 shows that the sample displayed sufficient variation on all measures in the present study. Internal consistency was excellent for all scales. Internal consistency was not presented for PDS cumulative trauma as this is a checklist of traumatic events. Participants had an average age of 47.34 (S.D. = 10.82). There were 71 males, 34 females, and one participant with unreported gender. Based on responses to the PDS, 96% of participants reported posttraumatic stress symptoms in the moderate range or above, and 74% met full DSM-IV criteria for a diagnosis of PTSD, suggesting that the present sample had high levels of posttraumatic stress.

Regarding depression, 83% of participants had PHQ-9 scores indicative of at least moderate depression, with 51% of participants reporting severe depression.

While relevant norms and descriptors were not available for the CTQ, 93% of the sample reported experiencing some childhood trauma on the CTQ.

### Cross-Site Comparison

As data were collected from 4 different sites, four one-way analyses of variance were conducted to assess for potential group differences on predictor and outcome variables. No significant difference between sites was found for depression, *F*(3,101) = .23, *p* = .88, *η*^*2*^ = .01. Similarly, there were no significant differences between sites for posttraumatic stress symptoms, *F*(3,102) = .33, *p* = .80, *η*^*2*^ = .01) or cumulative trauma, *F*(3,102) = .80, *p* = .50, *η*^*2*^ = .02). However, there was a significant difference between sites for childhood trauma, *F*(3,102) = 3.37, *p* = .02., *η*^*2*^ = .10). Post hoc comparisons revealed that participants from the community addictions service had significantly lower childhood trauma (M = 37.67; S.D. = 14.85) than the inpatient addiction service (M = 60.67; S.D. = 17.03) and the psychological therapies service (M = 60.96; S.D. = 28.63).

### Correlational Findings

Correlations between cumulative and childhood trauma, total trauma appraisals and subscales, posttraumatic stress, and depression symptoms are presented in Table 2. Cumulative trauma was weakly positively correlated with total trauma appraisals, the individual appraisal scales of alienation and shame, and depression symptoms. Notably, cumulative trauma was not significantly correlated with posttraumatic stress symptoms. Childhood trauma showed small positive correlations with fear appraisals, anger appraisals and posttraumatic stress symptoms, and showed moderate correlations with total TAQ appraisals, alienation, shame, betrayal and self-blame appraisals, and depression.

**Table 2:** First order correlations (r) between childhood and cumulative trauma, trauma appraisals (TAQ and subscales), posttraumatic stress (PDS) and depression (PHQ-9)

	1	2	3	4	5	6	7	8	9	10
1. PDS cumulative trauma	–									
2. CTQ childhood trauma	.26*	–								
3. TAQ total	.20*	.45**	–							
4. TAQ alienation	.25*	.35**	.82**	–						
5. TAQ fear	.12	.21*	.86**	.72**	–					
6. TAQ shame	.20*	.44**	.89**	.70**	.68**	–				
7. TAQ self-blame	.16	.42**	.75**	.50**	.51**	.68**	–			
8. TAQ betrayal	.13	.49**	.67**	.45**	.41**	.61**	.45**	–		
9. TAQ anger	.09	.23*	.68**	.45**	.62**	.54**	.32**	.30**	–	
10. PDS symptoms	.10	.24*	.67**	.66**	.63**	.57**	.44**	.39**	.43**	–
11. PHQ depression	.23*	.33**	.69**	.69**	.59**	.61**	.46**	.40**	.46**	.60**

* p < .05. ** p < .01

Total TAQ scores, and alienation, fear and shame appraisals showed strong positive correlations with PDS symptoms and PHQ-9, whilst self-blame, betrayal and anger appraisals showed moderate positive correlations with these outcome variables.

### Mediation Analyses

#### Mediation Models with Total TAQ Scale as Mediators

**Fig. 1:**
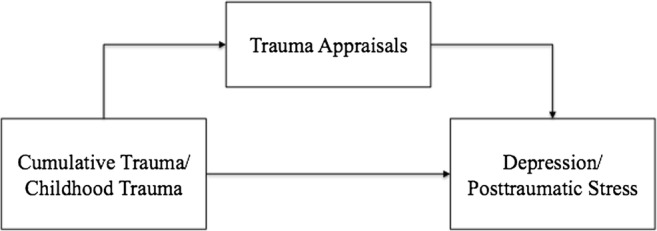
Conceptual mediation model showing both predictor and outcome variables, with full TAQ scale as mediator

Three of four hypothesised models were tested initially. No model was tested for cumulative trauma to posttraumatic stress symptoms, as these variables were uncorrelated. Models 2, 3 and 4 were tested, with trauma appraisals as sole mediator between, 2) cumulative trauma and depression; 3) childhood trauma and posttraumatic stress symptoms and 4) childhood trauma and depression. Figure 1 shows a conceptual diagram of these models.

Table 3 shows results for models 2, 3 and 4. In model 2, significant total effects were found between cumulative trauma and depression, and when full trauma appraisal questionnaire scores were entered as a mediator of this relationship, a significant indirect effect was observed. The direct effect was no longer significant, indicating full mediation and suggesting that the relationship between cumulative trauma and depression is mediated by trauma appraisals.

**Table 3:** Summary of mediation models 2, 3 and 4 for cumulative trauma and childhood trauma

Model	Independent Variable (IV)	Mediating Variable (MV)	Dependent Variable (DV)	Effect of IV on M	Effect of M on DV	Direct Effect	Indirect Effect	95% CI	Total Effect
2	Cumulative Trauma	TAQ	PHQ-9	.21*	.67**	.08	.14*	(.03–.27)	.22*
3	Childhood Trauma	TAQ	PDS symptoms	.45**	.66**	−.05	.30**	(.18–.46)	.24*
4	Childhood Trauma	TAQ	PHQ-9	.45**	.66**	.02	.30**	(.18–.44)	.32**

* p < .05. ** p < .01

In model 3, the relationship between childhood trauma and posttraumatic stress was significant. When appraisals were entered as a mediator, a significant indirect effect was observed and the direct effect was no longer significant, indicating full mediation.

Finally, for model 4, the relationship between childhood trauma and depression was significant, but when appraisals were entered as a mediator, this relationship was no longer significant. A significant indirect effect via appraisals was observed, suggesting full mediation.

#### Mediation Models with TAQ Subscales as Mediators

The above models were then tested again, but with all six trauma appraisal subscales as mediators.

#### Model 2 Extended: Cumulative Trauma & Depression

Table 4 shows the results for cumulative trauma as a predictor of depression when all six TAQ subscales were entered as parallel mediators. There was a significant total effect. The only significant indirect effect was observed for alienation appraisals, suggesting that alienation appraisals mediate the relationship between cumulative trauma and depression when appraisals of fear, shame, self-blame, betrayal and anger are controlled for. The direct effect was not significant, suggesting full mediation via alienation appraisals.

**Table 4:** Summary of extended model 2; parallel mediation model for cumulative trauma

Independent Variable (IV)	Mediating Variable (MV)	Dependent Variable (DV)	Effect of IV on M	Effect of M on DV	Direct Effect	Indirect Effect	95% CI	Total Effect
Cumulative Trauma	TAQ alienation	PHQ-9	.26**	.45**	.05	.12*	(.05–.24)	.22*
	TAQ fear		.13	.05		.01	(−.02–.08)	
	TAQ shame		.20*	.12		.02	(−.03–.10)	
	TAQ self-blame		.16	.07		.01	(−.01–.07)	
	TAQ betrayal		.15	.02		.00	(−.02–.04)	
	TAQ anger		.11	.12		.01	(−.01–.07)	

* p < .05. ** p < .01

#### Model 3 Extended; Childhood Trauma and Posttraumatic Stress

Results for childhood trauma as a predictor of posttraumatic stress with all six trauma appraisal subscales as mediators are shown in Table 5. Total effects were significant, and the only significant indirect effect was found for alienation appraisals. The direct effect was not significant when mediators were included. These findings suggest that alienation appraisals fully mediate the relationship between childhood trauma and posttraumatic stress when appraisals of shame, fear, anger, betrayal and self-blame are simultaneously controlled for.

**Table 5:** Summary of extended models 3 and 4; parallel mediation models for childhood trauma

Independent Variable (IV)	Mediating Variable (MV)	Dependent Variable (DV)	Effect of IV on M	Effect of M on DV	Direct Effect	Indirect Effect	95% CI	Total Effect
Childhood Trauma	TAQ alienation	PDS symptoms	.34**	.41**	−.01	.14*	(.05–.27)	.24*
	TAQ fear		.20*	.25		.05	(.00–.16)	
	TAQ shame		.44**	−.04		−.02	(−.15–.10)	
	TAQ self-blame		.42**	.07		.03	(−.05–.13)	
	TAQ betrayal		.50**	.08		.04	(−.07–.16)	
	TAQ anger		.23*	.05		.01	(−.03–.07)	
	TAQ alienation	PHQ-9	.34**	.42**	.04	.14*	(.06–.27)	.31**
	TAQ fear		.20*	.06		.01	(− .03–.09)	
	TAQ shame		.44**	.14		.06	(−.05–.18)	
	TAQ self-blame		.42**	.08		.03	(−.03–.11)	
	TAQ betrayal		.50**	−.01		.00	(−.10–.09)	
	TAQ anger		.23*	.11		.03	(−.01–.09)	

* p < .05. ** p < .01

#### Model 4 Extended; Childhood Trauma and Depression

Table 5 shows results for individual appraisal scales as mediators of the relationship between childhood trauma and depression. Again, significant total effects were observed, with alienation appraisals acting as the only significant mediator. There was no significant direct effect when appraisal scales were entered as mediators, suggesting that alienation appraisals fully mediate the relationship between childhood trauma and depression, when controlling for other appraisal types.

## Discussion

Cumulative and childhood trauma have both been posited as risk factors for the development of PTSD in adulthood and the current study sought to assess whether these proposed relationships could be explained via the mediating effect of trauma appraisals. Results indicate that alienation appraisals fully mediate the relationships between childhood trauma and symptomatology. This suggests that a focus on the manner in which individuals appraise their trauma, specifically in terms of alienation, disconnection and loneliness, should be a core component in the treatment of trauma-related distress.

Significant relationships were found between childhood trauma and both depression and posttraumatic stress in adulthood. Similarly, a significant positive correlation was found between cumulative trauma and depression. Notably however, the relationship between cumulative trauma and posttraumatic stress was not significant. When total appraisals of trauma (as indicated by the TAQ) were tested as mediators of the above relationships, full mediation was indicated in all cases. Trauma appraisals mediated the relationship between cumulative trauma and depression, between childhood trauma and depression, and between childhood trauma and posttraumatic stress. In support of the first hypothesis, this suggests that participants who had experienced higher levels of childhood trauma tended to appraise traumatic events more negatively, and suffered greater psychological distress as a result, in the form of posttraumatic stress and depression. The second hypothesis was partially supported, in that participants with higher levels of cumulative trauma also showed an elevated tendency to make negative appraisals of traumatic events, which led to higher levels of depression, but not higher levels of posttraumatic stress.

When differing types of trauma appraisal were considered simultaneously, only appraisals of alienation mediated the relationships between cumulative/childhood trauma and depression/posttraumatic stress.

Taken together, the findings are supportive of the general cognitive appraisal model of PTSD (e.g. Ehlers and Clark 2000), in that prior experiences and characteristics of trauma influence the manner in which the worst traumatic event is appraised, which determines the extent of negative psychological consequences. Consistent with prior meta-analytic findings (Brewin et al. 2000), this pattern was found for childhood trauma and posttraumatic stress symptoms. The present research also extended this to considering depression as a further marker of trauma-related distress. However, whilst cumulative trauma had an indirect effect on depression through trauma appraisals, there was no direct or indirect effect on symptoms of posttraumatic stress through appraisals. This is only partially supportive of Gamache-Martin et al. (2013), who found that cumulative trauma was predictive of both depression and posttraumatic stress. This finding may be due to measurement differences in cumulative trauma. The current study used total number of different trauma types indicated on the PDS to quantify cumulative trauma, whereas Gamache-Martin et al. (2013) used cumulative indices of low, medium and high betrayal. These indices were not used in the current study as it was intended as a test of the appraisal model more generally, without specifically focusing on betrayal trauma theory. While Wilker et al. (2015) support measuring cumulative trauma as number of trauma types, it should be noted that their study used a checklist of 62 traumatic events, of which some were specifically tailored to the post conflict Ugandan population in their study. The current study used the 12-item checklist on the PDS, which has not been empirically validated for this purpose. This relatively constricted range of potential trauma types, coupled with the lack of specific items tailored to the Northern Ireland context may have limited the validity of this measure in the current study. Nevertheless, the current study is the first to our knowledge to demonstrate the mediating role of trauma appraisals between cumulative and childhood trauma, and markers of trauma-related distress in treatment seeking adults.

Alienation emerged as the only significant mediating appraisal when all appraisals were simultaneously controlled for. This supports prior findings from DePrince et al. (2011) wherein alienation was consistently related to PTSD and depression across a range of samples, and was the only appraisal type that predicted all three forms of trauma related distress in their analyses (PTSD symptoms, depression and dissociation). Additionally, DePrince et al. (2011) found that alienation was associated with trauma-related distress even when social support was controlled for, suggesting that it is not merely an indicator of social support. DePrince et al. (2011) suggested that a sense of disconnectedness from self and others might contribute to diverse forms of distress. It is possible that childhood abuse and neglect may lead to difficulties in connecting or relating to self/others, which may in turn foster appraisals of alienation when traumatic events are experienced, ultimately resulting in trauma-related distress. Ehlers and Clark’s (2000) cognitive model provides a useful explanation of the cognitive maintenance factors in PTSD, but does not elaborate on the manner in which prior experiences may prime or sensitize individuals toward making negative appraisals when traumatic events are experienced. Attachment theory may provide a useful bridge to this end, with insecure attachment being a likely contributor to later appraisals of alienation. Indeed, fearful attachment predicts lifetime PTSD symptoms (O'Connor and Elklit 2008), and insecure adult attachment mediates the relationship between childhood trauma and somatizing symptoms in women (Waldinger et al. 2006).

Present findings suggest that alienation appraisals are key drivers of symptomatology, and alienation has previously been identified as a potential barrier to exposure therapy for PTSD (Ehlers et al. 1998). Ebert and Dyck (2004) argued in a review paper that alienation is a feature of mental defeat, and that this is likely to undermine exposure therapy by both preventing the formation of an effective working relationship, and by negatively biasing the client’s expectations for therapy. They suggest that treatments that focus on identity, in addition to the rebuilding of meaningful interpersonal interactions, may be beneficial. Taken together, these findings suggest that assessment and intervention of trauma related difficulties should place strong emphasis on alienation.

Additionally, as cumulative trauma and PTSD symptoms were not correlated, this suggests an optimistic picture whereby cumulative trauma does not necessarily cause PTSD, rather the appraisal process is key. As appraisals are amenable to intervention, this provides hope for future treatments.

A final implication is that focused intervention on appraisals of alienation may reduce symptoms of both posttraumatic stress and depression. It may therefore be important to assess whether a client presenting with depression is experiencing this as a result of how they are appraising prior trauma. A thorough evaluation of prior traumatic events should be part of the routine assessment of depression.

A number of limitations were evident in the current study. It is possible that the indirect effects observed solely through alienation appraisals may result from overlap with items on the PDS. Several items in the avoidance subscale of the PDS symptom scale appear similar to alienation appraisals on the TAQ, for example ‘feeling distant or cut off from people around you’, ‘feeling emotionally numb (for example being unable to cry or have loving feelings)’. There are no items on the PDS that correspond as closely with shame, self-blame or betrayal appraisals. However, there are a number of items that relate directly to anger and fear, but anger and fear appraisals were not significant mediators when considered alongside alienation appraisals, reinforcing the primacy of alienation appraisals. It is also possible that differing appraisals may predict other markers of trauma-related distress stress not measured in the current study, such as dissociation or substance use. Moreover, it is possible that additional forms of appraisal unaccounted for by the TAQ, or other un-assessed variables may be responsible for the observed relationships. A concurrent analysis of additional mediating variables, additional markers of trauma-related distress, and the specific effects of distinct trauma types may be useful in clarifying the role of appraisals of trauma.

Data were unavailable on ethnicity; however, the Northern Irish population is highly racially homogenous, with only 1.8% belonging to a non-white ethnic minority (NISRA 2012) which may limit representativeness. The study did not control for concurrent substance use, which might be expected to be higher in the addiction service group, nor was stage of therapy controlled for. Additionally, while participants from the community addictions site had significant levels of childhood trauma, they had significantly lower childhood trauma than those from the inpatient unit and the psychological therapies site. The models tested could not account for this difference, representing a limitation of the study. However, analyses demonstrated no significant differences on core outcome variables across sites.

A further limitation surrounds the use of a cross-sectional methodology. The present indirect effect models cannot indicate causality of relationships. Prospective longitudinal studies examining childhood trauma, trauma exposure and appraisals and later development of posttraumatic stress are required to strengthen causal conclusions. The current study can however provide support for the empirical plausibility of the indirect paths from experiencing trauma, to trauma related distress, via the appraisal process. The present findings are supportive of the core process of negative appraisal of traumatic events and sequalae emphasised in cognitive models of PTSD (Ehlers and Clark 2000). Moreover, they support Ehlers and Clark’s (2000) contention that prior experiences (in this case, childhood trauma) would increase the propensity to negatively appraise traumatic experiences, leading to symptoms of PTSD. However, in contrast to prior research, cumulative trauma was not related to posttraumatic stress severity.

The finding that alienation appraisals were the only significant mediator of the relationships between childhood trauma and posttraumatic stress/depression suggests that negative early experiences may influence the way in which individuals relate to themselves and others, thereby increasing risk for negative appraisals and subsequent distress in later life. The current findings imply that therapeutic approaches that focus on the negative appraisal process in PTSD are supported, and suggest that particular focus should be directed toward appraisals of alienation and disconnectedness.
